# Automated Detection of Sickled Erythrocytes in Peripheral Blood Smear Images Using YOLO-Based Object Detection

**DOI:** 10.21203/rs.3.rs-10580495/v1

**Published:** 2026-09-04

**Authors:** Juhair Fiaz Alam, Linh Nguyen T Tran, Daniel Agrawal, Abrar Faiaz Bari, Reagan Phariss, Karla Mercedes Paz Gonzalez, Gift Modekwe, Lukman Tijani, Qiugang Lu, Jenifer Gomez-Pastora

**Affiliations:** 1 Department of Computer Science, Texas Tech University, Lubbock, TX 79409, USA; 2 Department of Chemical Engineering, Texas Tech University, Lubbock, TX 79409, USA; 3 Department of Medical Oncology, Division of Hematology and Medical Oncology, Texas, Tech University Health Sciences Center, Lubbock, TX 79409, USA

**Keywords:** Sickle cell disease, YOLO, Peripheral blood smear, Red blood cell morphology, Biomedical image analysis, Machine learning

## Abstract

Sickle cell disease (SCD) is characterized by abnormal red blood cell (RBC) morphology that drives hemolysis and microvascular complications, yet manual smear evaluation remains subjective and difficult to standardize in heterogeneous fields. In this work, we present a clinically grounded and reproducible framework for cell-level detection of sickled erythrocytes using You Only Look Once (YOLO)-based object detection. The pipeline integrates controlled sample dilution before imaging to reduce cell crowding, an overlapping tiling strategy to preserve morphological detail, and a standardized annotation protocol to minimize ambiguity in borderline cells. Models were trained on peripheral blood smear images from both SCD patients across distinct clinical states, including steady-state and vaso-occlusive crisis. Our results show that YOLO11 achieved the highest performance of mAP50–95 = 0.857, with minimal inter-class confusion. Error analysis indicates that most misclassification arises from background regions and annotation-driven exclusions, highlighting the influence of data quality and labeling strategy on model performance. These findings demonstrate accurate cell-level detection performance of our method in complex smear images and establish a scalable framework for automated RBC morphological analysis, with potential relevance for disease monitoring and deployment in resource-limited settings pending further validation.

## 1. Introduction

Hemoglobinopathies are inherited disorders caused by genetic mutations that alter the structure or production of hemoglobin and collectively represent one of the most common monogenic disease groups worldwide [1], [2], [3]. Among these, sickle cell disease (SCD) is a prototypical structural hemoglobinopathy arising from a point mutation in the β-globin gene that produces hemoglobin S (HbS). Under deoxygenated conditions, polymerization of HbS increases red blood cell (RBC) rigidity and promotes morphological deformation, ultimately contributing to hemolysis and microvascular obstruction [4], [5].

Peripheral blood smear microscopy provides a direct and widely accessible means of visualizing these morphological changes at the single-cell level. Importantly, RBC populations in SCD exhibit substantial morphological heterogeneity, including fully sickled cells, partially deformed or elongated cells, and morphologically near-normal erythrocytes within the same field of view. This variability across disease states is illustrated in Figure 1, which presents representative blood smear images from non-severe, severe, and vaso-occlusive crisis conditions. This heterogeneity reflects dynamic physiological conditions such as oxygen tension and intracellular hemoglobin composition and complicates consistent visual assessment [5], [6]. In routine practice, manual smear review is labor-intensive and subject to inter-observer variability, particularly when distinguishing intermediate or borderline morphologies and when cells overlap or cluster [6]. The coexistence of multiple RBC morphologies within a single field inherently limits image-level classification approaches. In contrast, object detection frameworks enable simultaneous localization and classification of individual cells, making them well-suited for analyzing complex peripheral blood smears.

From a clinical and translational perspective, reliable identification and quantification of sickled erythrocytes are relevant not only for diagnosis, but also for disease monitoring and evaluation of therapeutic response. However, the global burden of SCD is highest in regions where access to specialized hematology expertise and advanced diagnostic infrastructure is limited [3], [7]. Even in well-resourced settings, manual microscopy does not scale efficiently for high-throughput analysis or large retrospective studies. These challenges highlight the need for automated, image-based approaches that can objectively detect and localize individual sickled erythrocytes from standard microscopy images. Such methods have the potential to reduce subjectivity, improve reproducibility, and enable scalable morphological analysis across diverse clinical and research environments [6], [7].

Recent advances in deep learning have enabled automated analysis of hematological images with performance approaching expert-level interpretation in tasks such as cell detection, classification, and segmentation [11], [12], [13]. Convolutional neural network (CNN)-based methods have been successfully applied to RBC morphology analysis, including the identification of sickle cells in peripheral blood smears [14], [15], [16], [17]. However, many existing studies rely on image-level classification or patch-based approaches, which assume relatively homogeneous cell populations and fail to capture the spatial distribution, overlap and coexistence of multiple cell morphologies within a single field of view [15], [16], [18], [19], [20]. Object detection frameworks address these limitations by simultaneously localizing and classifying individual cells, enabling direct cell-level quantification in complex and densely populated microscopic images. Among these, the YOLO family has emerged as a leading architecture due to its single-stage design, high inference speed, and robust performance in detecting small objects across diverse imaging modalities [21]. YOLO formulates detection as a unified regression problem that directly predicts bounding box coordinates and class probabilities from the full image in a single forward pass. Its computational efficiency makes the framework particularly well-suited for high-throughput biomedical imaging applications [21].

In biomedical microscopy, YOLO-based models have demonstrated strong performance in tasks such as malaria parasite identification and histopathological cell localization [22], [23], [24], [25]. These studies demonstrate YOLO’s capability to accurately detect small and visually similar objects under variable imaging conditions, a challenge that closely parallels the identification of sickled and non-sickled erythrocytes in peripheral blood smears. In parallel, YOLO-based approaches have been applied to general blood cell detection and counting, with models such as SDE-YOLO, YOLO-BC, and multiple YOLO variants (v5 through v10) benchmarked for detecting RBCs, white blood cells (WBCs), and platelets [26], [27], [28], [29]. However, these studies target morphologically typical cell populations and do not address disease-specific morphologies, such as sickled and partially deformed cells in sickle cell anemia. This limitation has motivated the development of object detection frameworks specifically tailored to SCD-relevant morphologies, including recent studies on YOLO-based automated sickle cell detection [8], [9], [10]. Early implementation using YOLOv3 primarily focused on binary slide-level screening (sickle positive vs. negative), rather than multi-class, cell-level detection of both normal and sickled RBCs within heterogeneous fields of view [8]. More recent work has proposed an optimized YOLOv8-based model (DREPADetect), reporting 87.6% precision, 87.6% recall, and 64.6% mAP50–95 on a dataset containing normal, sickle, and other cell types [9]. Another YOLOv8n-based approach reported lower class-specific performance, achieving 76.4% precision, 88.6% recall, and 60.1% mAP50–95 for the sickle cell class, highlighting ongoing challenges related to overlap and ambiguous morphology in crowded smear images [10]. Despite these advances, existing YOLO-based studies for sickle cell detection remain limited by inconsistencies in dataset quality, lack of clinical stratification, and incomplete reporting of sample preparation, microscopy acquisition, and annotation protocols [8], [9], [10]. As a result, reported performance varies widely, and reproducibility and direct comparison across studies remain challenging. Furthermore, some studies focus on binary classification or do not explicitly address the morphological heterogeneity and cell overlap characteristic of SCD peripheral blood smears. Collectively, these limitations highlight the need for clinically grounded, reproducible detection pipelines that integrate standardized experimental design with robust object detection models, as pursued in this study.

In this study, we present a clinically grounded and reproducible framework for cell-level detection based on the YOLO (You Only Look Once) algorithm that emphasizes a clear and reproducible end-to-end methodology spanning sample preparation, microscopy imaging, annotation, and model training; including a controlled strategy to reduce cell crowding and mitigate overlaps within the field of view. While work has been done before in the area of YOLO-based sickle cell detection (SCD) [8], [9], [10], these studies face limitations in dataset quality, detection accuracy, and reproducibility. More specifically, a key distinguishing aspect of this study is the use of high-quality peripheral blood smear images obtained from both steady-state sickle cell patients and patients experiencing acute vaso-occlusive crisis. Unlike many existing datasets that lack detailed clinical stratification, our dataset captures physiologically relevant variations in RBC morphology across distinct disease states. Moreover, controlled sample dilution reduces cell crowding, while an overlapping tiling strategy preserves fine morphological detail that would otherwise be lost during image downscaling. By incorporating images from both baseline and crisis conditions, our dataset provides a clinically representative foundation for training and evaluating detection models and enables improved generalization across the full morphological spectrum encountered in practice. Using this framework, we systematically evaluate multiple modern YOLO models and demonstrate improved detection performance relative to previously reported approaches. Beyond performance gains, this study introduces a standardized and transparent framework spanning sample preparation, annotation, preprocessing, and model evaluation that supports reproducibility and enables more reliable comparison across future studies.

## 2. Results

### 2.1. Model scaling comparison

To compare performance across model architectures and scales, 15 models were evaluated, from the YOLOv5, YOLOv8, and YOLO11 families using mAP50–95. Figure 2 presents their performance across five model sizes for each model family. YOLO11l achieved the highest performance, reaching a peak mAP50–95 of 0.857. This was followed by YOLOv8s at 0.850 and YOLOv5l at 0.835 within their respective families. Across all model families, increasing model size did not consistently improve performance, as, in several cases, extra-large variants performed similarly to or worse than their corresponding large or medium models. This behavior may reflect diminishing performance gains with increasing model complexity and greater tendency toward overfitting, particularly given the relatively limited dataset size. Based on these results, YOLOv5l, YOLOv8s, and YOLO11l, which achieved the highest mAP50–95 within their respective model families, were selected for detailed evaluation.

### 2.2. Detailed model performance

Table 1 presents the detailed performance of YOLOv5l, YOLOv8s, and YOLO11l across precision, recall, F1-score, mAP50, and mAP50–95 for both overall and class-specific results. Across all classes, YOLOv5l, YOLOv8s, and YOLO11l had mAP50–95 values of 0.835, 0.850, and 0.857, respectively. The mAP50 values were consistently high across all models, with 0.962 for YOLOv5l, 0.975 for YOLOv8s, and 0.977 for YOLO11l. At the individual class level, YOLOv8s achieved the highest mAP50–95 for the SS class at 0.857, while YOLO11l achieved the highest mAP50–95 for the RBC class at 0.859. Recall and precision values were high across all models, with precision ranging from 0.928 to 0.955 and recall from 0.929 to 0.945. The F1-scores across all classes were 0.950 for YOLOv5l, 0.939 for YOLOv8s, and 0.929 for YOLO11l.

Although YOLOv5l achieved the highest overall F1 score (0.950), the differences in F1 score among the three models were relatively small. Model selection was therefore based primarily on mAP50–95, which provides a more comprehensive assessment of object detection performance by incorporating both classification accuracy and bounding box localization quality across multiple IoU thresholds [35], [36], [37]. YOLO11l achieved the highest mAP50–95 value (0.857), indicating more consistent detection and localization performance than other evaluated models. Consequently, YOLO11l was selected as the representative model for subsequent confusion matrix and qualitative analyses. The strong performance of YOLO11l may reflect architectural improvements introduced in newer YOLO model families, including refinements in feature extraction and detection head design [21], [31].

Accordingly, Figure 3 shows the confusion matrix for YOLO11l. The model correctly classified 308 RBC and 84 SS, with minimal misclassification between the two classes. Only one RBC instance was misclassified as SS and two SS instances were misclassified as RBC. Most errors were related to background predictions, where 12 RBC and 3 SS instances were missed, and some background regions were incorrectly predicted as RBC (20) or SS (12).

### 2.3. Qualitative results

Figure 4 compares a representative blood smear image, showing ground truth annotations (Figure 4a) and the corresponding YOLO11l predictions (Figure 4b), including the confidence scores for each detected object. The model accurately localizes and classifies RBC and SS instances, with predicted bounding boxes closely matching the ground truth annotations. Most detections exhibit high confidence scores (~0.9), with only one lower-confidence score (~0.7). Three unannotated cells are visible in Figure 4a, which were intentionally excluded from annotation due to ambiguous morphology. Of these, two cells were correctly left unassigned to any class by the model, consistent with the annotation criteria shown in Figure 4b. One of these cells exhibited elongated morphology but lacked clearly defined pointed ends. The third unannotated cell was incorrectly predicted by the model as an RBC, which represents a false positive. Overall, qualitative results show strong agreement between predictions and ground truth annotations, with only limited discrepancies primarily associated with unannotated cells.

## 3. Discussion

The results of the model scaling comparison demonstrate a consistent trend in which newer YOLO architectures outperform earlier versions in overall detection performance. In particular, YOLO11l achieved the highest mAP50–95, indicating improved detection and localization performance across multiple IoU thresholds, a metric commonly used to assess detection quality in object detection frameworks [38], [39], [40], [41], [42]. This distinction is particularly important in biomedical imaging applications, where accurate boundary localization is critical for downstream quantitative analysis.

Across all model families, increasing model size did not consistently improve performance. In several cases, extra-large variants performed similarly to or worse than their corresponding large or medium models, suggesting diminishing performance gains with increasing model complexity. The lack of consistent improvement in extra-large variants may reflect mild overfitting, particularly given the relatively limited dataset size and controlled imaging conditions. These findings further indicate that mid-to-large architectures may provide an optimal balance between detection performance and computational efficiency.

Analysis of the confusion matrix reveals that many errors arise from detections involving unlabeled or ambiguously labeled regions rather than direct confusion between RBC and SS classes. The low rate of inter-class misclassification indicates that the model effectively captures distinguishing morphological features of fully sickled erythrocytes. The remaining errors primarily involve missed labeled instances (false negatives) or detections of unlabeled cells (false positives).

These errors are closely linked to the annotation strategy employed in this study. Representative examples illustrating the influence of annotation ambiguity on apparent false-positive detections are shown in Figure 5. Cells with ambiguous morphology, partial visibility, or imaging artifacts were intentionally excluded from annotation to maintain label clarity and consistency. As a result, some apparent false positives may correspond to biologically relevant cells that were deliberately omitted from the ground truth dataset, suggesting that some detections may reflect morphological features not represented in the annotation set. Qualitative prediction analysis (Figure 4) further supports this interpretation, as the model frequently refrained from assigning class labels to ambiguous cells while maintaining accurate classification of clearly defined RBC and SS morphologies.

Beyond model performance, these findings highlight the importance of morphological heterogeneity in peripheral blood smears from patients with SCD. The strong detection performance and low inter-class confusion indicate that the model captures key geometric characteristics of fully sickled erythrocytes, including elongated shape, pointed ends, and reduced symmetry, even in densely populated fields of view.

The presence of borderline morphologies underscores the continuous nature of RBC morphological transitions in SCD rather than a strictly binary classification. These findings suggest that automated detection frameworks may offer a more consistent and scalable approach to morphological assessment, particularly when intermediate phenotypes complicate visual interpretation. Furthermore, the ability of the model to distinguish fully sickled cells in heterogeneous and overlapping fields suggests that geometric features may provide substantial discriminatory power for automated classification, even in the absence of additional biophysical measurements.

In addition to biological heterogeneity, the strong performance observed in this study was also influenced by experimental design and preprocessing strategy. The controlled sample dilution strategy reduced cell crowding and overlap, improving both annotation accuracy and detection reliability. In addition, the use of an overlapping tiling approach preserved fine morphological details that would otherwise be lost during direct image downscaling, which is critical for distinguishing subtle differences between cell classes. The observed performance improvements relative to previously reported YOLO-based sickle cell detection studies [9], [10] suggest that data quality and preprocessing strategy play an important role in model performance. In addition, the dataset exhibits class imbalance between RBC and SS populations, which may influence sensitivity toward the majority class. Although high performance was observed across both classes, future studies should evaluate class balancing strategies or weighted loss functions to further assess robustness and minimize potential classification bias.

From a translational perspective, reliable cell-level detection of sickled erythrocytes from standard peripheral blood smear images may support applications in automated screening, quantitative disease monitoring, and evaluation of therapeutic response in patients undergoing transfusion or disease-modifying treatment. Such approaches may be particularly valuable in settings where access to expert hematological assessment is limited. Once sufficiently validated, automated morphology analysis frameworks may help reduce reliance on specialized expertise while enabling scalable and accessible image-based assessments across diverse clinical environments.

Some limitations should be considered when interpreting these findings. First, the dataset size is relatively limited, which may affect generalizability despite the use of data augmentation and tiling strategies. In particular, the controlled acquisition conditions may not capture variability in imaging hardware or sample preparation encountered in real clinical settings. Second, the annotation framework simplifies RBC morphology into two classes and does not fully capture the continuum of morphological states observed in SCD. Third, manual annotation introduces potential subjectivity, particularly in borderline cases where classification criteria may be less well-defined. Finally, all images were acquired under controlled experimental conditions, and model performance may differ under more variable clinical imaging environments.

Future work should extend this framework toward multi-class morphological classification, enabling differentiation between partially sickled, irreversibly sickled, and other abnormal RBC phenotypes. Incorporating quantitative morphological descriptors or integrating detection outputs with complementary biophysical measurements may further strengthen the relationship between image-based features and clinical disease severity. In addition, approaches such as consensus annotation or probabilistic labeling could be explored to better represent morphological uncertainty in ground truth data. Validation using larger and more diverse multi-center clinical cohorts will be essential to establish generalizability and support potential clinical translation.

In this work, we developed a clinically grounded and reproducible framework for cell-level detection of sickled erythrocytes in peripheral blood smear images using modern YOLO-based object detection models. By integrating controlled sample preparation, a morphology-preserving tiling strategy, and a standardized annotation protocol, we demonstrated that high detection accuracy can be achieved even in heterogeneous and densely populated smear images. Importantly, our findings highlight that detection performance is not solely dependent on model architecture, but is strongly influenced by data quality, preprocessing strategy, and annotation design.

Beyond model performance, this study underscores the potential of automated cell-level analysis to provide consistent and scalable assessment of RBC morphology in SCD. Following validation in larger and independent cohorts, such approaches may support applications in diagnostic screening, disease monitoring, and evaluation of therapeutic response, where the burden of SCD is highest and access to specialized hematological expertise is constrained. Overall, this work establishes a robust foundation for automated sickled erythrocyte detection and provides a pathway toward more comprehensive clinically accessible morphological analysis.

## 4. Methods

This section describes the complete end-to-end framework for cell-level detection of sickled erythrocytes, encompassing sample preparation, microscopy acquisition, annotation, preprocessing, and model training, as illustrated in Figure 6. Each stage of the pipeline was designed to preserve native RBC morphology and ensure reproducibility across experimental and computational steps.

### 4.1. Data collection and sample preparation

Peripheral whole blood samples were collected from patients with confirmed SCD in ethylenediaminetetraacetic acid (EDTA) anticoagulant tubes following informed consent, in accordance with the Institutional Review Board (IRB)-approved protocol of the Texas Tech University Health Sciences Center (Protocol number IRB: FY2025–228). RBCs were isolated from whole blood by centrifugation, plasma removal, and washing of the cellular fraction three times with phosphate-buffered saline (PBS). Each washing step consisted of centrifugation at 2,000 × *g* for 5 min, followed by removal of the supernatant and resuspension of the RBC pellet in fresh PBS. This procedure removed plasma components and residual anticoagulants prior to smear preparation. To reduce cell crowding and minimize overlap within the microscopic field of view, washed RBC suspensions were diluted in PBS at a calculated ratio immediately prior to smear preparation. This controlled dilution improved spatial separation between cells, thereby enhancing annotation accuracy and reducing ambiguity in object detection. Diluted samples were gently mixed by slow pipette aspiration and dispensing to ensure homogeneous cell distribution without inducing mechanical deformation. Thin blood smears were prepared by placing a small volume of the diluted RBC suspension onto clean glass microscope slides and spreading the sample using a standardized wedge-smear technique. Prepared smears were imaged without chemical staining to preserve native RBC morphology. Blood smear images were acquired using optical microscopy with an EVOS^™^ M5000 Imaging System (Thermo Fisher Scientific, Waltham, MA, USA) equipped with a 60X objective, producing images at a resolution of 2048 × 1536 pixels under consistent illumination and acquisition settings across all samples.

### 4.2. Annotation technique

All microscopic images were manually annotated using the open-source tool LabelImg, which provides an interactive graphical interface for drawing bounding boxes and assigning class labels to objects of interest [30]. Annotation was performed at the single-cell level, where each visible RBC was localized using a rectangular bounding box and assigned to its corresponding morphological class. In this study, morphologically near-normal and partially deformed erythrocytes were grouped under the RBC class, whereas fully sickled erythrocytes exhibiting clearly defined elongated, crescent, or pointed-end morphologies were assigned to the sickle cell (SS) class. Annotations were created by visually inspecting each image and drawing tight bounding boxes around individual RBCs. Cells that were partially visible at the image boundary or affected by significant artifacts were excluded from annotation to avoid introducing ambiguous labels. Only well-resolved cells with clearly identifiable morphology were included in the training and evaluation datasets. Annotations were exported in YOLO format, with bounding box coordinates normalized relative to image dimensions. This format is directly compatible with the YOLO training pipeline and enables efficient loading and preprocessing of labeled data during model training. To improve annotation consistency, all images were labeled following a unified annotation protocol that defined the criteria for each morphological class. Representative examples were established prior to annotation to minimize subjectivity, particularly for intermediate or borderline cell morphologies. When necessary, annotations were reviewed and refined to ensure labeling accuracy and consistency across the dataset. This annotation strategy enables precise localization and classification of individual RBCs within full-field blood smear images, providing the required ground truth for training and evaluating the proposed YOLO-based object detection framework.

### 4.3. Preprocessing and tiling strategy

The annotated dataset consisted of 141 peripheral blood smear images, containing 1,410 RBC-class cells and 376 SS-class cells. Annotation files generated in XML format were parsed to extract bounding box coordinates and class labels, which were subsequently converted into the normalized text format required for YOLO-based training. The dataset was randomly divided into training and validation subsets using an 80:20 split, resulting in 113 training images and 28 validation images, with no overlap between sets.

Given the high spatial resolution of the microscopy images, an additional preprocessing stage was required prior to model training. The original microscopy images had a resolution of 2048 × 1536 pixels, which substantially exceeded the standard input dimensions typically used for YOLO-based object detection frameworks. Direct downsampling of the full-resolution images would reduce fine morphological features critical for distinguishing sickle erythrocytes from morphologically near-normal RBCs. Therefore, to preserve local cellular morphology while maintaining compatibility with the detection framework, an overlapping image tiling strategy was implemented prior to model training.

Under this strategy, each microscopy image, denoted as $\text{I}\in R^{\left(H\times W\right)}$, where *H* and *W* represent image height and width, respectively, was subdivided into smaller overlapping image tiles of dimensions *T*_*H*_ × *T*_*W*_. Adjacent tiles were generated using a fixed overlap distance *a* to minimize information loss and reduce partial object truncation near tile boundaries. The tile positions were adjusted to maintain consistent tile dimensions near the edges, while remaining fully contained within the original image boundaries. Bounding box annotations from the original microscopy image were assigned to a tile only when the complete object was fully enclosed within the tile boundaries. Subsequently, the bounding box coordinates were adjusted relative to the tile origin coordinates (*x*_0_, *y*_0_) to generate tile-level annotations compatible with YOLO training. Tiles containing no valid bounding boxes were excluded from training to ensure that the training dataset primarily consists of informative image regions containing annotated cellular structures.

Meanwhile, a total of 547 tiled images were generated from the original 141 microscopy images using a tile size of 1,024 × 768 pixels with a 150-pixel overlap between adjacent tiles. This preprocessing strategy preserved high-resolution morphological information while simultaneously increasing the effective number of training samples available for model optimization.

A detailed description of the tiling implementation is provided in Supplementary Information (Section S1 and Algorithm S1).

### 4.4. YOLO detection framework

The YOLO framework treats object detection as a unified regression problem, where it directly predicts bounding box coordinates and class probabilities from images in a single forward pass. Unlike traditional multi-stage detection pipelines that rely on region proposals and separate classification stages, YOLO employs a single CNN to perform detection end-to-end, which enables efficient and real-time inference. The model divides an input image into a grid structure, where each grid cell is responsible for predicting a fixed number of bounding boxes, including confidence scores and class probabilities. This way, the entire image is processed simultaneously with global context, which reduces background false positives and improves robustness across diverse imaging conditions. Its unified design allows the model to be trained directly on detection objectives while maintaining high computational efficiency [21]. In this work, several modern YOLO variants for implementation were evaluated, as described below.

### 4.5. Training setup

In this work, several modern YOLO variants were evaluated, including YOLOv5, YOLOv8, and YOLO11. For each model family, five different model sizes were trained on our dataset: nano (n), small (s), medium (m), large (l), and extra-large (x), corresponding to progressively increasing network capacity and parameter count (Supplementary Table S1). In total, 15 models were evaluated. All models were implemented using the Ultralytics framework and initialized with pretrained weights on the COCO dataset [31], [32].

Models were trained on NVIDIA T4 or A100 GPUs, depending on availability, using a consistent set of hyperparameters to maintain methodological consistency. Hardware variation did not affect the model configuration, training procedure, or evaluation protocol. Although the original microscopy images had a resolution of 2048 × 1536 pixels, an overlapping tiling strategy was used to preserve local morphological details while generating smaller image regions for training. These tiles were subsequently resized to 1024 × 1024 pixels during training to ensure compatibility with YOLO architectures, which operate most efficiently on input dimensions that are multiples of 32. Caching was also enabled to load data faster during training. A batch size of 8 was used, and each model was trained for up to 150 epochs. To prevent overfitting and reduce unnecessary computation, early stopping was applied with a patience of 40 epochs. All other training parameters were kept at their default values as defined by the Ultralytics implementation [31], [33]. These settings were selected to provide stable and consistent performance, based on the results of multiple preliminary experiments with different training configurations. A custom data augmentation strategy was used during training, which includes, but is not limited to, mosaic augmentation, horizontal flipping with a fixed probability, random scaling and translation, as well as photometric transformations using HSV (hue, saturation, and value) adjustments. These augmentations helped the models become more robust to varying cell morphology, imaging conditions, and overlapping cell detection.

### 4.6. Evaluation Metrics

Model performance was evaluated using standard object detection metrics, including precision, recall, F1 score, and mean average precision (mAP). Precision and recall were used to evaluate the detection performance of the model in terms of false positives and false negatives. Precision (*P*) is calculated as the number of true positives (*TP*) divided by the sum of true positives and false positives (*FP*). Recall (*R*) is calculated as the number of true positives divided by true positives plus false negatives (*FN*).


(1) $$P=\frac{TP}{TP+FP}$$

(2) $$R=\frac{TP}{TP+FN}$$

Intersection over union (IoU) was used to determine whether a prediction is a true positive. It is calculated as the area of intersection between the true bounding box and the model predicted bounding box, divided by the area of their union.


(3) $$IoU=\frac{\text{Area of Intersection}}{\text{Area of Union}}$$

Increasing the confidence threshold for predictions generally increases precision while decreasing recall [32], [34], reflecting the standard precision-recall tradeoff in object detection tasks. Consequently, the F1 score, defined as the harmonic mean of precision and recall, is commonly used to balance these competing metrics. The F1 score decreases substantially when either precision or recall is disproportionately low.


(4) $$F1=\frac{2PR}{P+R}$$

In contrast to the F1 score, which evaluates performance at a selected confidence threshold, mean average precision summarizes performance across the precision-recell curve. mAP50 is calculated at an IoU threshold of 0.50, whereas mAP50–95 averages performance across IoU thresholds ranging from 0.50 to 0.95 in increments of 0.05.

(5) $$mAP50=\frac{1}{n}\sum_{i=1}^{n}AP_{i}\;\text{for}\;n\;\text{classes}$$

where *AP*_*i*_ represent the average precision for class *i*, and *n* is the total number of classes. Average precision is computed as the area under the precision-recall curve for each class. mAP50–95 extends this metric by averaging mAP values across IoU thresholds ranging from 50% up to 95%.

mAP50–95 was used as the primary metric for model comparison because it reflects both detection accuracy and bounding box localization quality across multiple IoU thresholds. This evaluation protocol follows a standard object detection benchmark established in the Microsoft COCO dataset [32].

## Supplementary Material

Supplementary Files

This is a list of supplementary files associated with this preprint. Click to download.

- Supplementary.docx

## Figures and Tables

**Figure 1:**
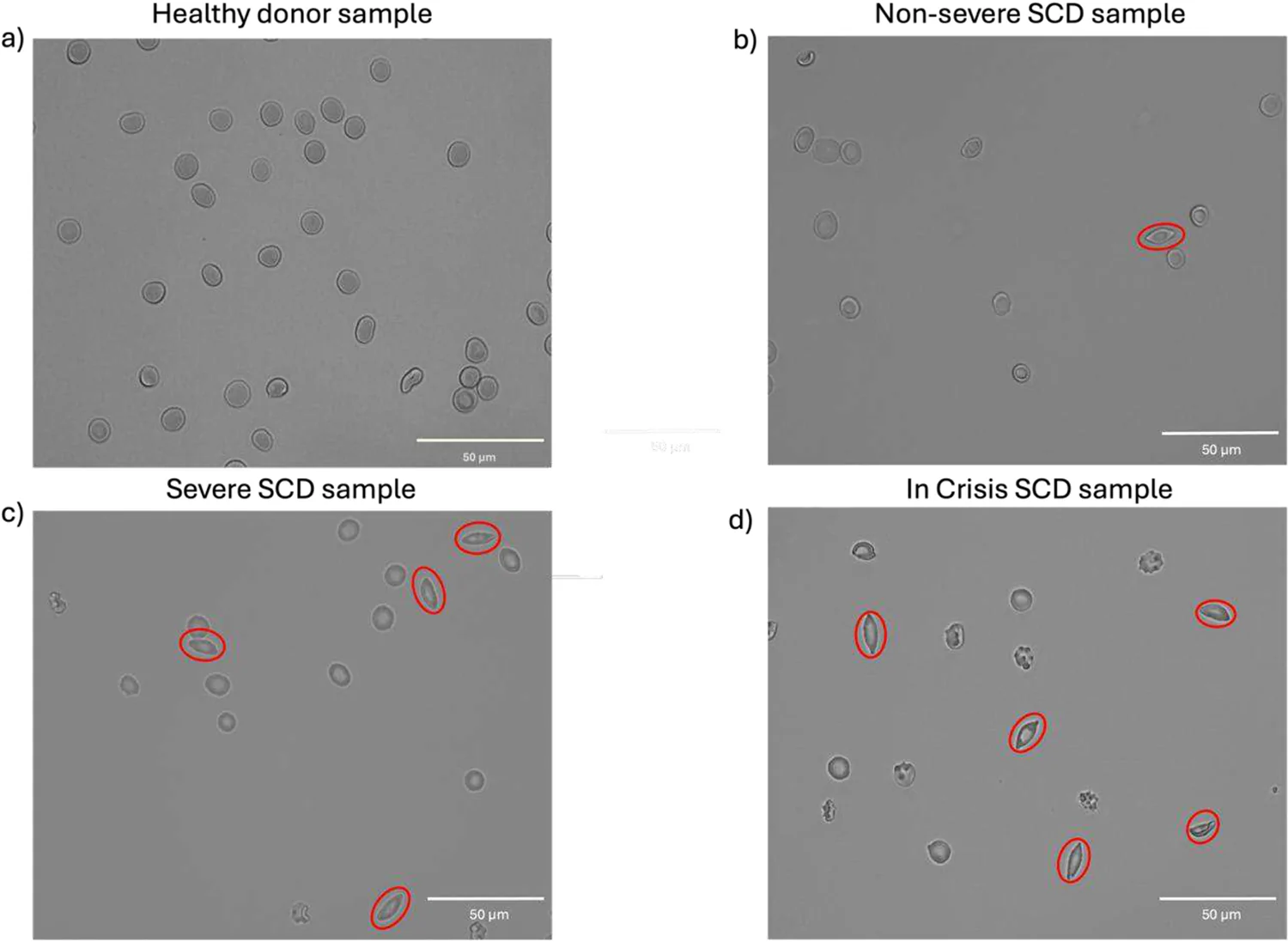
Representative peripheral blood smear images illustrating morphological variability of red blood cells (RBCs) across sickle cell disease (SCD) conditions: (a) healthy donor, (b) non-severe SCD, (c) severe SCD, and (d) vaso-occlusive crisis (VOC). Sickled erythrocytes exhibiting elongated or crescent morphology are highlighted (red circles), demonstrating increased prevalence and morphological severity across disease states. Scale bars: 50 μm.

**Figure 2.**
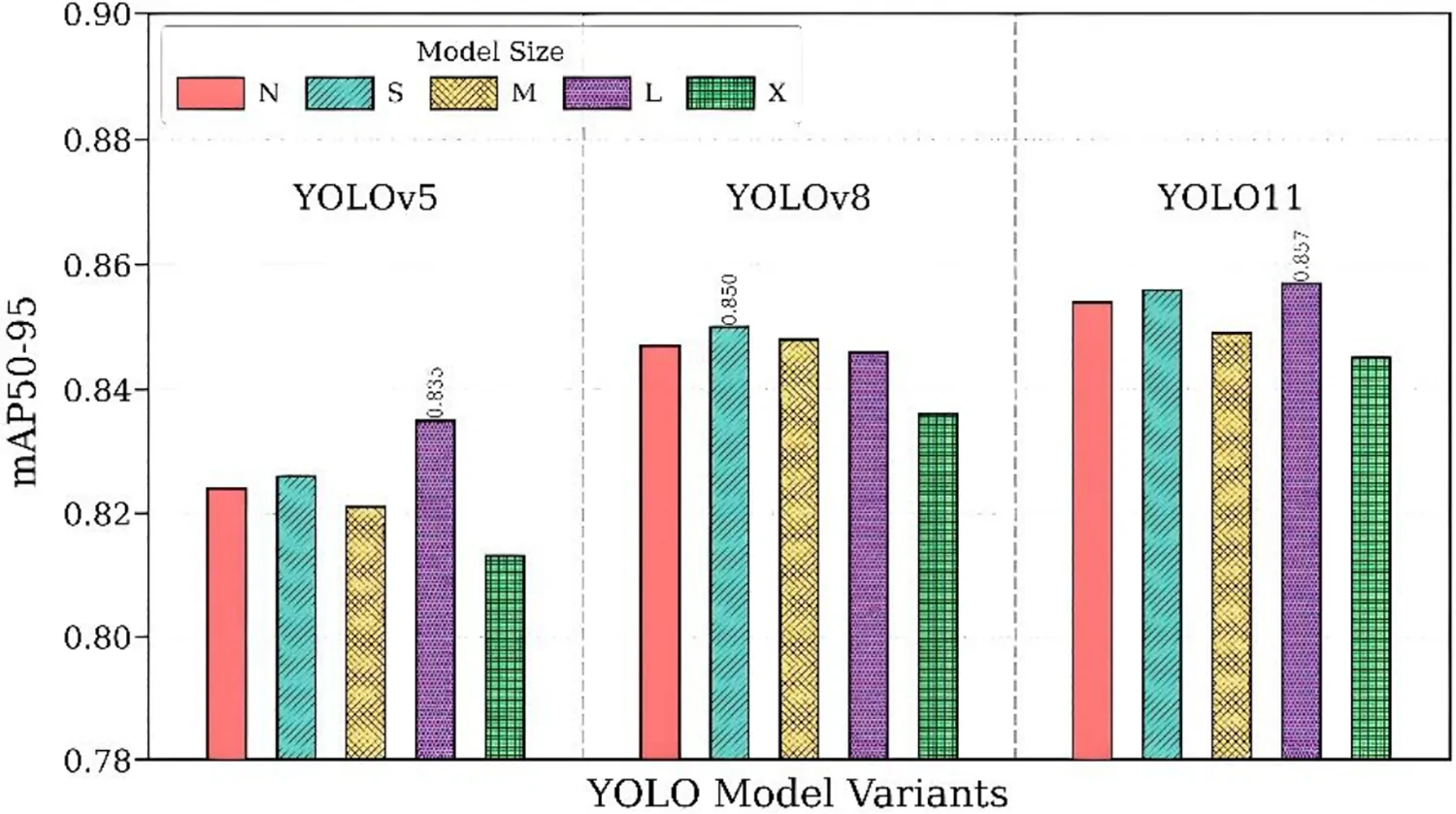
mAP50–95 performance of YOLOv5, YOLOv8, and YOLO11 across model scales (nano, small, medium, large, extra-large). YOLO11l achieves the highest performance (0.857), while increasing model size does not consistently improve performance across architectures.

**Figure 3.**
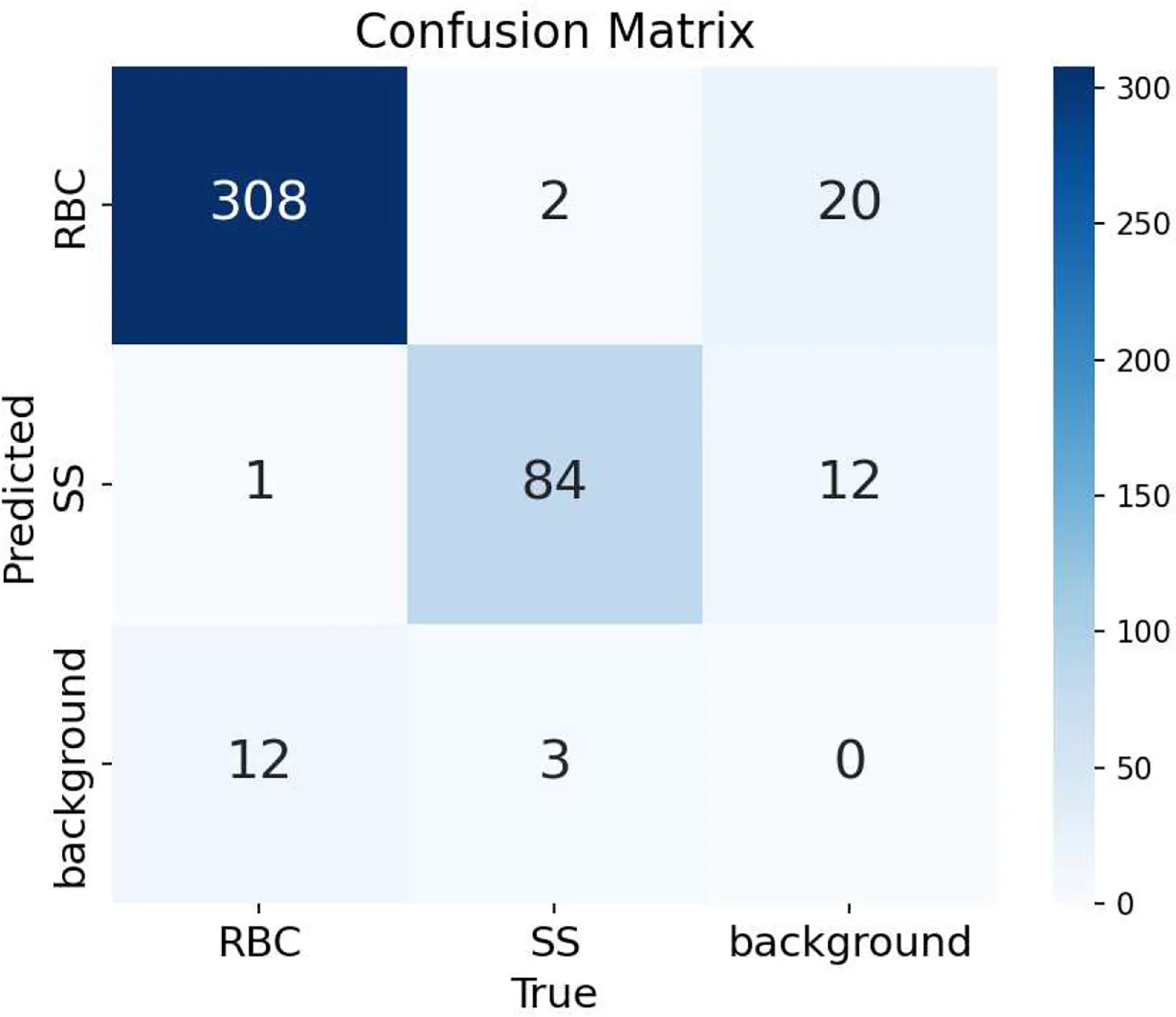
Confusion matrix for YOLO11l.

**Figure 4.**
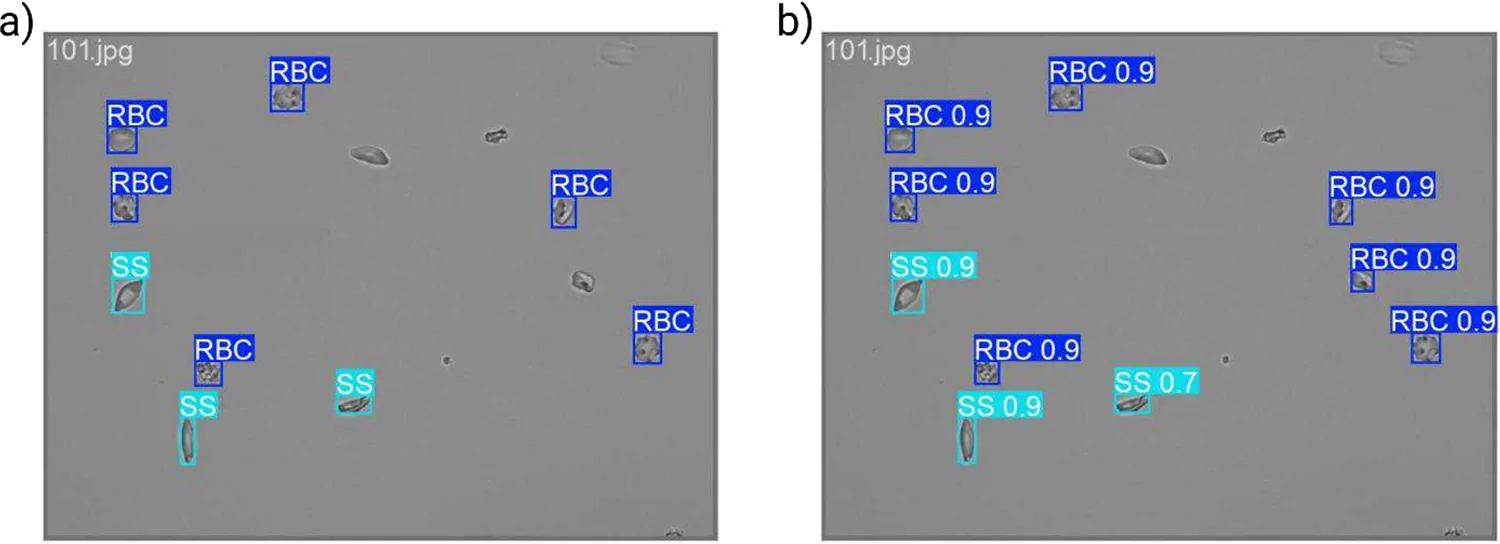
Qualitative comparison between ground truth annotations (a) and YOLO11l predictions (b) for a representative peripheral blood smear image. RBC and SS detections are shown with confidence scores, demonstrating strong agreement between predicted and annotated cell instances.

**Figure 5.**
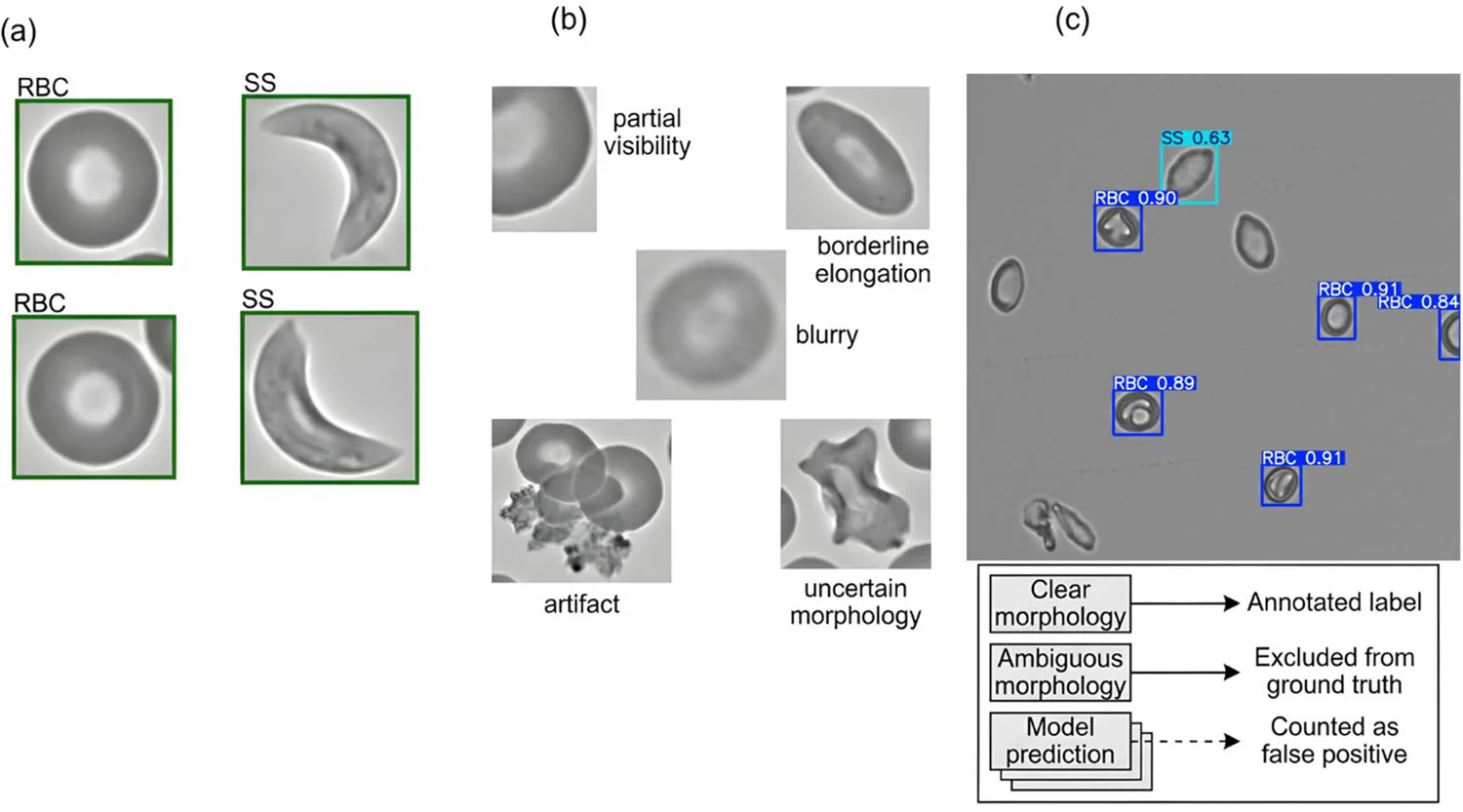
Influence of annotation ambiguity on apparent false-positive detections. (a) Representative examples of manually annotated RBC and SS morphologies used for YOLO model training. (b) Examples of excluded erythrocyte morphologies and imaging conditions that were intentionally omitted from annotation. (c) Representative YOLO model detections on morphologically ambiguous or excluded cellular structures. Blue bounding boxes indicate RBC predictions, while cyan bounding boxes indicate SS predictions.

**Figure 6.**
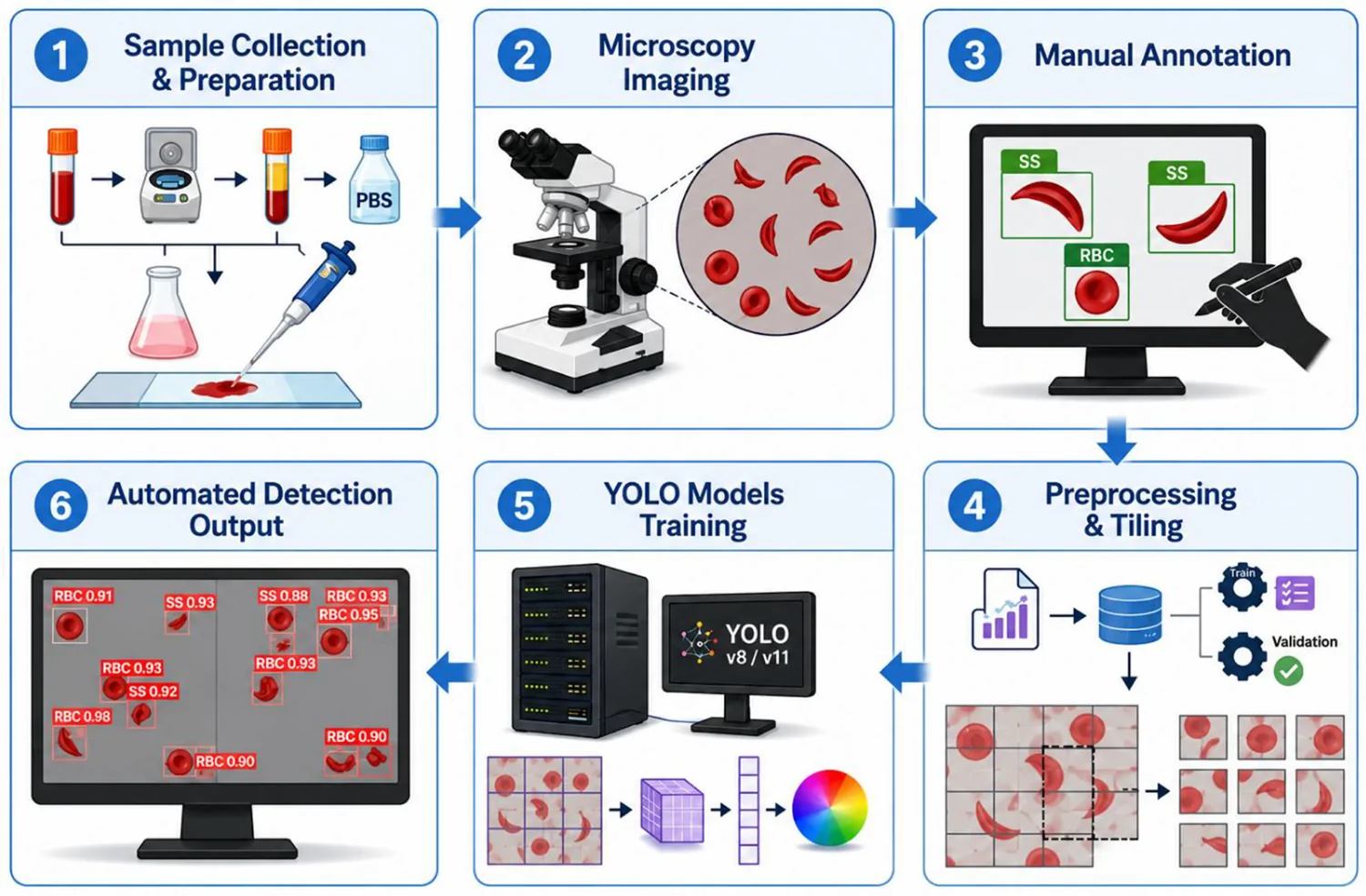
End-to-end methodology pipeline for YOLO-based sickle cell detection

**Table 1.** Detection performance of YOLOv5l, YOLOv8s, and YOLO11l across all classes and individual RBC and SS classes. Metrics include precision, recall, F1-score, mAP50, and mAP50–95, highlighting differences in classification and localization performance across models.

Model	Class	Precision	Recall	F1	mAP50	mAP50-95
**YOLOv5l**	All	0.955	0.945	0.950	0.962	0.835
	RBC	0.943	0.969	0.956	0.954	0.830
	SS	0.967	0.921	0.943	0.970	0.840
**YOLOv8s**	All	0.940	0.938	0.939	0.975	0.850
	RBC	0.938	0.953	0.945	0.979	0.843
	SS	0.943	0.922	0.932	0.970	0.857
**YOLO11l**	All	0.928	0.929	0.929	0.977	0.857
	RBC	0.955	0.931	0.943	0.983	0.859
	SS	0.902	0.926	0.914	0.971	0.854

## Data Availability

The datasets generated and/or analyzed during the current study are not publicly available due to Institutional Review Board (IRB) restrictions and patient privacy considerations but are available from the corresponding author upon reasonable request, subject to institutional approval.
